# Transcriptomic Profiling of Peripheral B Cells in Antibody Positive Sjogren’s Patients Reveals Interferon Signature

**DOI:** 10.3390/genes15050628

**Published:** 2024-05-15

**Authors:** Mehrnaz Maleki-Fischbach, Kelsey Anderson, Evans R. Fernández Pérez

**Affiliations:** 1 Division of Rheumatology, National Jewish Health, Denver, CO 80206, USA; 2 Center for Genes, Environment, and Health, National Jewish Health, Denver, CO 80206, USA; andersonk@njhealth.org; 3 Division of Pulmonary, Critical Care and Sleep Medicine, National Jewish Health, Denver, CO 80206, USA

**Keywords:** Sjögren’s disease, B cells, gene expression

## Abstract

Background: Sjögren’s disease (SjD) is a common systemic autoimmune disease that affects mainly women. Key pathologic features include the infiltration of exocrine glands by lymphocytes and the activation of B lymphocytes with the production of autoantibodies. We aimed to analyze the transcriptome of circulating B cells from patients with SJD and healthy controls to decipher the B-cell-specific contribution to SJD. Methods: RNA from peripheral blood B cells of five untreated female patients with SjD and positive ANA, positive anti-SSA (both Ro-52 and Ro-60), positive anti-SSB and positive rheumatoid-factor, and five healthy controls was subjected to whole-transcriptome sequencing. A false discovery rate of < 0.1 was applied to define differentially expressed genes (DEG). Results: RNA-sequencing identified 56 up and 23 down DEG. Hierarchal clustering showed a clear separation between the two groups. Ingenuity pathway analysis revealed that these genes may play a role in interferon signaling, chronic mycobacterial infection, and transformation to myeloproliferative disorders. Conclusions: We found upregulated expression of type-I and type-II interferon (IFN)-induced genes, as well as genes that may contribute to other concomitant conditions, including infections and a higher risk of myeloproliferative disorders. This adds insight into the autoimmune process and suggests potential targets for future functional and prognostic studies.

## 1. Introduction

Sjögren’s is a chronic systemic autoimmune disease characterized by inflammation of the salivary and lacrimal glands. It predominantly affects women, with a female-to-male sex ratio of 9:1. Lymphocytic infiltration of the secretary glands leads to organ dysfunction, often with ocular and oral dryness, known as sicca syndrome. Extraglandular manifestations can present as hematological, pulmonary, renal, vascular, musculoskeletal, neurological, and cutaneous involvement.

The etiology of Sjögren’s disease remains unclear, but current data suggest that it is caused by multiple factors, including genetic and epigenetic changes. The activation of B lymphocytes has been proposed as one of the key drivers of disease. In Sjögren’s disease, B lymphocytes are responsible for various findings, including hypergammaglobulinemia and the presence of autoantibodies, including anti-SSA/Ro60/TROVE2 (in 60–80% of patients), anti-SSB/La48 (in 30–40% of patients) [1] and anti-SSA/Ro52/TRIM21 (in 60–70% of patients) [2,3]. Patients with Sjögren’s disease are at high risk of developing lymphoproliferative disorders, particularly non-Hodgkin B cell lymphomas. The presence of lymphoid germinal centers on biopsy may predict the transformation to lymphoma, or more commonly, mucosa-associated lymphoid tissue (MALT) lymphomas [3,4].

Previous work on gene expression profiling in patients with Sjögren’s disease demonstrated a type-I and type-II interferon signature in peripheral blood mononuclear cells (PBMCs), salivary gland tissues, and peripheral CD19+ B cells. Transcriptome profiling of B cells has also shown upregulation of CX3CR1, a regulatory factor in B cell malignancies, as well as several members of the TNF superfamily. Downregulated genes include suppressors of cytokine signaling [5]. In this study, we applied RNA-sequencing in a comprehensive analysis of the whole transcriptome of peripheral blood B cells from patients with Sjögren’s disease. The objective of this study was to gain a better understanding of the regulatory molecular pathways in B cells.

## 2. Materials and Methods

### 2.1. Patients

Consecutive new patients were identified in the Rheumatology Clinic at National Jewish Health. Five patients fulfilling the American European Consensus Group criteria for the diagnosis of Sjögren’s disease and five healthy controls provided by the “National Jewish Health Program in Mucosal Inflammation and Immunity Human Blood Preparation Consortium” were included (Table 1). The Institutional Board Review of National Jewish Health approved the study (HS2888). All patients and controls gave their consent. All subjects were female: one Caucasian of European ancestry, one Caucasian of North African ancestry, and three Hispanic/Latinas of Central–South American ancestry (mean age 41.4 ± 12.2). All patients had positive serology for ANA, anti-SSA (both Ro-52 and Ro-60), anti-SSB, and rheumatoid factor, and were negative for β₂ macroglobulin and cryoglobulin screen. All subjects were without immunomodulatory therapy at the time of sampling. We assessed each patient’s EULAR Sjögren’s Syndrome Disease Activity Index (ESSDAI) and EULAR Sjögren’s Syndrome Patient Reported Index (ESSPRI).

### 2.2. RNA Extraction and Sequencing

Blood samples were collected and PBMCs were immediately isolated through centrifugation. B-cells were negatively selected via magnetic separation using the StemCell Technologies EasySep Human B-cell enrichment kit per the manufacture’s protocol. Total RNA from B-cells was extracted using the mirVana miRNA isolation kit. The quantity and quality of RNA were assessed using Qubit 2.0 and Bionalalyzer, respectively. We used the KAPA Stranded mRNA-Seq kit (Wilmington, MA, USA) to build whole polyA-selected RNA transcriptome libraries prior to sequencing. Samples were sequenced as barcoded pools in conjunction with HiSeq 2500 (Illumina; San Diego, CA, USA) to a depth of ~30 million reads per sample. as routinely performed by the National Jewish Health Genomics Facility.

### 2.3. Data Analysis

Adapters and reads with lengths less than 18 base pairs were removed using Skewer 0.2.2 [6]. Raw FASTQ reads were generated using the Illumina pipeline CASAVA V1.8.4, and the quality of the reads was assessed using FastQC (version 0.11.5) [7]. Using STAR aligner (version 2.4.1d) [8], we mapped the reads to the canonical chromosomes of the hg19 assembly of the human genome using gene annotations from Ensembl version 75 [9]. The featureCounts program from the Subread software package (v1.5.2) [10] was used to quantify the number of reads per gene that were used as input to DESeq2 (version 1.81) [11] to identify differentially expressed genes between Sjögren’s disease and control samples. *p*-values reported were adjusted for multiple testing using the method developed by Benjamini and Hochberg [12], and *p* < 0.1 was used as a cutoff. Principal components analysis (PCA) on the read counts per gene was performed using the prcomp function in R version 3.3.2. Hierarchical clustering and heatmap visualization were produced using the *clustermap* function of the Seaborn package (version 0.9.0) for Python (version 3.7) using Ward’s method for linkage on the Euclidean distances of the normalized counts.

Differentially expressed mRNAs in Sjögren’s disease subjects were compared to the cases described by Imgenberg-Kreuz J et al. [5], which were used as the validation cohort. The validation cohort [5] included 12 Caucasian women diagnosed with Sjögren’s disease, with a mean age of 61 and positive anti-SSA, as well as 20 B cell samples from healthy blood donors.

### 2.4. Functional Enrichment Analysis

Ingenuity pathway analysis was used to identify and interpret biologic pathways and diseases from the differentially expressed genes between subjects with Sjögren’s disease and control samples. The significance of the association between RNA transcripts and the canonical pathway was assessed using two criteria: (1) the ratio of the number of molecules mapping to the pathway and the total number of molecules involved in the canonical pathway; and (2) the Benjamini–Hochberg-corrected *p*-value from the right-tailed Fisher Exact test.

## 3. Results

We observed major differences in gene expression between subjects with Sjögren’s disease and healthy controls. At an FDR < 0.1 we observed a total of 79 differentially expressed genes; 56 upregulated, and 23 downregulated (Supplementary Table S1). Based on biological relevance, expression and significance, top genes associated with Sjögren’s disease and how they compared to published signatures are shown in Table 2.

**Figure 1:**
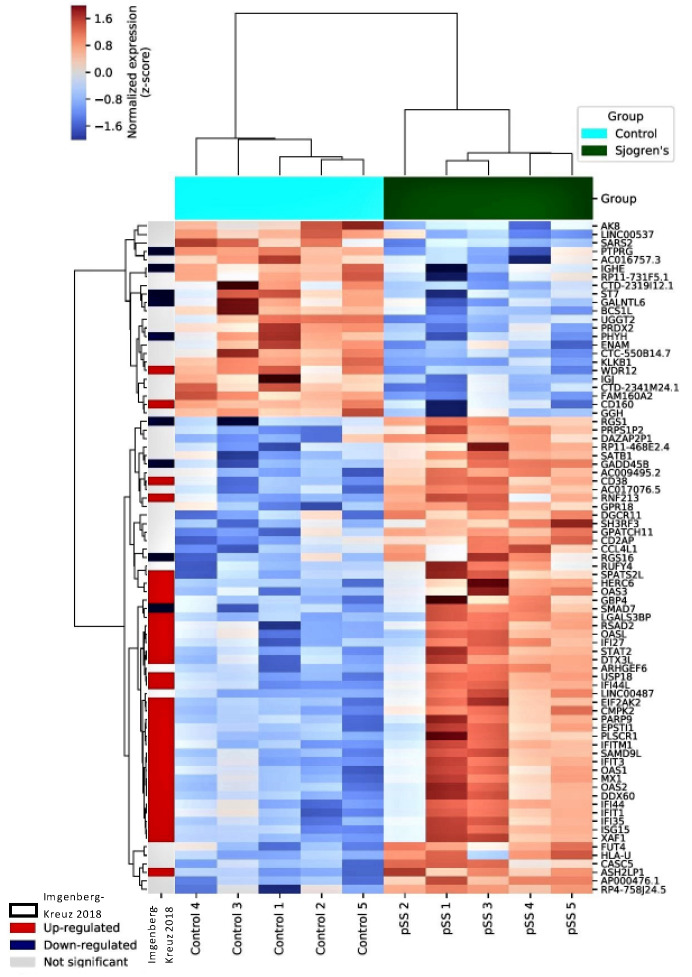
Clustering dendrogram of differentially expressed genes between Sjögren’s disease subjects and controls. The heatmaps show differentially expressed mRNAs in Sjögren’s disease subjects compared to controls and to Imgenberg-Kreuz J. et al. [5]. Samples were grouped using hierarchical clustering based on similar expression profiles. Heatmap color codes for column labels are indicated on the top right of the heatmap. The title of each label is displayed on the left side of each band. The data are represented by the Z-score of log_2_-normalized read counts. The color-key legend is shown on the top left of each heatmap: red (i.e., Z-score > 0) indicates over-expression; white indicates no change in gene expression; blue (i.e., Z-score < 0) indicates under-expression.

**Figure 2:**
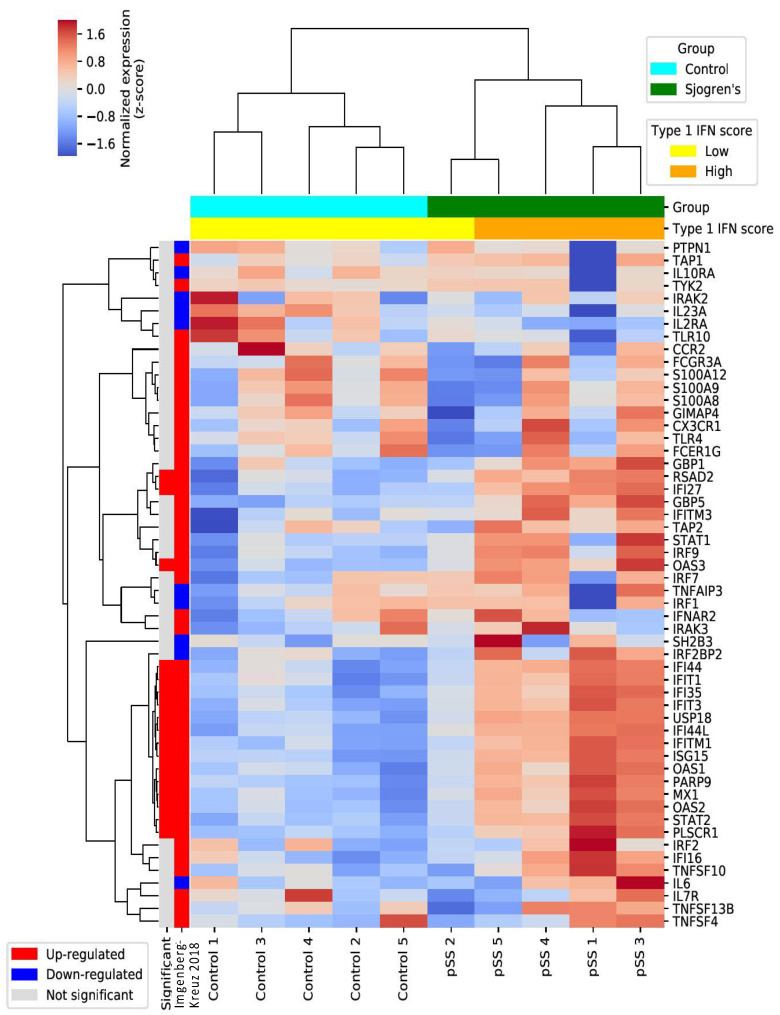
Clustering dendrogram of differentially expressed genes between Sjögren’s disease subjects and controls compared to validation cohort. This heatmap shows a set of interferon-related genes (selected by Imgenberg-Kreuz J. et al. [5]), distinguishing subjects from controls using unsupervised hierarchical clustering. The heatmap additionally overlays a type-I interferon signature defined by Imgenberg-Kreuz J. et al. based on the expression of IFI35, IFITM1, IRF7, MX1, and STAT1. Heatmap color codes for column labels are indicated on the bottom left of the heatmap.

Using normalized reads in conjunction with the Ward linkage clustering method, we performed unsupervised hierarchical clustering and observed a clear separation between the subjects (Figure 1 and Figure 2).

### Canonical Pathways

We used IPA to identify potential pathways affected by differentially expressed genes between the two groups. We found a total of nine pathways with –log (adjusted *p*-value > 1.36), including interferon signaling and pathways associated with mycobacterial infection (Table 3).

Further, we observed a total of 500 significant diseases and function annotations associated with these dysregulated genes (Supplementary Table S2).

## 4. Discussion

In the present study, we performed RNA-sequencing in five well-phenotyped patients with Sjögren’s disease who had not yet received any treatment, who tested positive for SSA (both Ro60 and Ro52), SSB, and rheumatoid factor, and compared these to five age- and sex-matched normal controls. The study cases are distinct from previous investigations, owing to the presence of antibody positivity for both SSA/Ro60 and Ro-52, SSB/La, and rheumatoid factor. It is noteworthy that SSA/Ro-52 is associated with more severe disease, as well as an increased risk of malignancies and interstitial lung disease [13,14,15]. A total of 79 genes were shown to be differentially expressed between the two groups, a set list of genes that involve seven pathways, including interferon signaling and receptors, in recognizing bacteria and viruses [16]. Compared to Imgenberg-Kreuz J et al. [5], who looked at gene expression difference in peripheral B cells in serologically positive Sjögren’s disease subjects and controls, 33 similar genes that matched significance and the trend of expression (Table 2) were seen, providing support for our data from an independent cohort.

The results of the canonical pathway analysis indicated “interferon signaling”, “pattern recognition receptors of bacteria and viruses”, “pyrimidine pathways”, and “interferon regulatory factors” to be the highest-ranking signaling pathways in our cases.

Type-I interferon signaling is a complex network of over 300 IFN-stimulated genes that encode many chemokines and cytokines [17]. These proteins are essential for the immune response and play an important role in host protection against pathogens and malignancies [18,19]. As therapeutics, they are also used to treat autoimmune disorders. The top differentially expressed genes also enriched on the “interferon signaling” in our dataset included IFIT (interferon-induced proteins with tetratricopeptide repeats) 3, IFIT1, OAS1, MX1, STAT2, IFI35, IFITM1, and ISG15. These genes are part of the interferon type-I signature in CD14 monocytes and were found to be upregulated in our subjects with Sjögren’s disease, which is consistent with the findings of Nezos et al. [20]. They observed upregulation of MX-1, IF44, IFI44L, and IFIT3 in the CD14 monocytes of 69 subjects with Sjögren’s compared to 44 healthy controls.

The IFIT genes play a critical role in the body’s anti-viral defense mechanism. We also found upregulation of MX-1, shown to act as a signal transducer and activator of transcription 2 (STAT2). The phosphorylation of STAT2 and STAT1 as a result of activated tyrosine kinase 2 (TYK2) and janus kinase 1 results in the formation of the STAT1/STAT2/IRF9 of INF-stimulated gene factor 3 (ISGF3) [21], which binds interferon-sensitive response elements (ISRE) [22]. STAT2-associating ISGF3 complexes play essential roles in immune responses, including the activation and propagation of immune cells, and inflammatory cytokine production and anti-viral signaling. It is important to note that STAT2 expression, which we observed to be notably increased in our Sjögren’s disease cases, has also been implicated in cancer development. Various experimental evidence strongly suggests that STAT2 plays a significant role in carcinogenesis, including lymphomas, which Sjögren’s disease patients are known to have a heightened risk of developing.

We found significant upregulation of the ubiquitin-like gene ISG15, which is among the most rapidly and strongly induced interferon-stimulated genes (ISGs). Recent research has revealed that the ISG15 protein can impede viral replication and modulate host immunity. Moreover, autophagy and regulation of the cancer microenvironment are some of the molecular processes in which ISG-15 is involved. Our research is in line with that of Cinoku et al. [23] In their study, they found that ISG-15 is elevated in both the labial minor salivary gland tissues and peripheral blood of patients with SS and lymphoma. Moreover, they observed that the levels of ISG-15 in labial minor salivary gland tissues are correlated with its levels in peripheral blood and extended the idea that ISG-15 may serve as a potential biomarker for Sjögren-related lymphoma development.

We also found enrichment of “pattern recognition receptors of bacteria and viruses” by OAS1, OAS2, and OAS3 genes. These genes encode an OAS enzyme family known to be vital in anti-viral responses, particularly OAS1 [24]. At the protein level, OAS1 risk variant has been found to be linked to decreased enzymatic activity in human peripheral blood mononuclear cells and viral clearance, which supports a potential role for defective viral infection resistance due to altered interferon response as a genetic pathophysiological basis of this complex autoimmune disease.

CMPK2 (cytidine/uridine monophosphate kinase) 2, significantly enriched in the “pyrimidine pathways” of our cases, is a type of thymidylate kinase that has been associated with mitochondrial DNA synthesis. In Sjögren’s disease, CMPK2 has been linked to the extent of immune cell activation and infiltration, as well as mitochondrial metabolic pathways that are believed to contribute to the pathogenesis of this disease [25]. Interestingly, adenylate kinase (AK) 8, which also serves in the “pyrimidine pathways”, was found to be downregulated. The AK family are essential enzymes that play a crucial role in maintaining the balance of adenine nucleotides within cells. It is critical for regulating various cellular processes, such as cell migration and differentiation. AK expression is downregulated in several tumors. Overexpression of AK in cancer cells has been linked with metabolic signaling, possibly resulting in unrestrained energy distribution in cancer cells [26].

The identification of EIF2AK2 in our dataset is consistent with a prior study that suggested that EIF2ZK2, along with LY6E, IL15, and CXCL10, might be used as the biomarkers for the treatment and diagnosis in Sjögren’s disease [27]. During innate immune signaling, EIF2AK2 inhibits the protein translation of inflammasome constituents and reduces inflammation.

We confirmed the distinct expression of important genes in B cells in a separate cohort, and our findings were consistent with previous studies. However, we must acknowledge that our study is constrained by the small sample size and the limited number of differentially expressed genes.

## 5. Conclusions

We found upregulated expression of interferon-induced genes, as well as genes that may contribute to other concomitant conditions, including a higher risk of myeloproliferative disorders. These findings provide insight into the autoimmune process and present promising avenues for future research in risk stratification and personalized therapeutic approaches. Indeed, stratifying patients with Sjögren’s disease based on the presence or absence of systemic manifestations, their serological immune profile, and their genetic profile, specifically between those with or without a predominant interferon gene or precancerous expression, could help evaluate the differentiated response to therapies in these subsets of patients with organ-specific involvement, and could also help enrich the design of future trials concerning Sjögren’s disease.

## Figures and Tables

**Table 1:** Clinical characteristics of the female patient cohort with Sjögren’s disease.

SjD Patients	Age at Onset	Age at B-Cells Sampling	+Abs	ESR mm/h	CRP mg/dL	IgG	SPEP	Cryoglobulin	ESSDAI	ESSPRI	Other Findings
SjD 1	20	26	ANA, Ro52, Ro60, SSB, RF	115	0.23	3142	Monoclonal IgG Kappa	0.1%	44	5.6	ILD/LIP, CNS, LAD, Low C4
SjD 2	29	33	ANA, Ro52, Ro60, SSB, RF	76	0.17	2480	Polyclonal gammopathy	0.0%	9	6	Fatigue, arthralgia, pLAD
SjD 3	36	42	ANA, Ro52, Ro60, SSB, RF	27	0.07	1633	Polyclonal gammopathy	0.1%	16	5.3	Fatigue, arthralgia, pLAD, airway disease
SjD 4	38	50	ANA, Ro52, Ro60, SSB, RF	41	0.21	1895	Normal	0.0%	4	5.6	Arthralgia, neutropenia, hypothyroidism
SjD 5	47	56	ANA, Ro52, Ro60, SSB, RF	33	0.13	2433	Polyclonal gammopathy	0.0%	16	25	Arthritis, lymphopenia, erythema, molitiforme

ANA, anti-nuclear antibody; CNS, central nervous system; CRP, c-reactive protein; Cryo, cryoglobulins; ESR, erythrocyte sedimentation rate; ESSDAI, EULAR Sjögren’s Syndrome Disease Activity Index; ESSPRI, EULAR Sjögren’s Syndrome Patient Reported Index; IgG, immunoglobulin G; ILD, interstitial lung disease; LIP, lymphocytic interstitial pneumonia; RF, rheumatoid factor; SPEP, serum protein electrophoresis, SjD, Sjögren’s disease; pLAD, palpable lymphadenopathy.

**Table 2:** Transcripts showing differential correlation between Imgenberg-Kreuz J et al. [5] and those replicated in the discovery dataset.

		Discovery Dataset *				Imgenberg-Kreuz J. et al. [5]			
Upregulated									
Gene ID	Gene Name	FPKM Cases	FPKM Control	Fold Change	q-Value **	FPKM Cases	FPKM Control	Fold Change	q-Value **
IFI27	Inferno α induced protein-27	3.96	0.10	36.81	1.22 × 10^−2^	8.20	0.10	88.24	3.75 × 10^−4^
IFI44	interferon induced protein 44	41.4	9.03	4.59	1.43 × 10^−2^	64.50	5.60	11.59	3.75 × 10^−4^
IFI44L	Interferon-induced protein 44-like	62.4	4.97	12.55	1.71 × 10^−5^	177.70	11.80	15.02	3.75 × 10^−4^
IFIT3	Interferon-induced protein with tetratricopeptide repeats 3	121.7	19.22	6.33	1.05 × 10^−3^	31.70	2.40	13.24	3.75 × 10^−4^
IFIT1	Interferon induced protein with tetratricopeptide repeats 1	42.59	7.75	5.50	4.46 × 10^−2^	21.20	0.90	23.39	3.75 × 10^−4^
MX1	MX dynamin like GTPase 1	121.6	37.89	3.21	1.28 × 10^−2^	145.70	34.20	4.26	3.75 × 10^−4^
IFI35	Interferon Induced Protein 35	49.81	31.57	1.58	4.71 × 10^−2^	27.70	9.60	2.89	3.75 × 10^−4^
STAT2	Signal transducer and activator of transcription 2	90.74	62.19	1.46	7.94 × 10^−2^	66.30	29.80	2.22	3.75 × 10^−4^
USP18	Ubiquitin specific peptidase 18	27.44	2.93	9.37	9.92 × 10^−6^	9.20	8.00	11.36	3.75 × 10^−4^
OAS1	2′-5′-oligoadenylate synthetase 1	132.9	54.63	2.43	4.71 × 10^−2^	67.30	10.20	6.57	3.75 × 10^−4^
OAS2	2′-5′-oligoadenylate synthetase 2	59.61	28.30	2.24	4.80 × 10^−2^	51.00	10.00	5.08	3.75 × 10^−4^
OAS3	2′-5′-oligoadenylate synthetase 2	16.98	3.86	4.39	6.22 × 10^−2^	14.20	3.70	3.85	3.75 × 10^−4^
OASL	2′-5′-oligoadenylate synthetase like	12.68	2.93	4.34	6.22 × 10^−2^	4.30	1.10	3.91	3.75 × 10^−4^
CMPK2	Cytidine/uridine monophosphate kinase 2—mitochondrial Interferon stimulated gene	8.23	0.59	13.85	1.37 × 10^−10^	23.30	2.20	10.62	3.75 × 10^−4^
LGALS3BP	Galectin 3 binding protein	34.43	6.03	5.71	1.31 × 10^−4^	13.70	2.10	6.63	3.75 × 10^−4^
SPATS2L	Spermatogenesis associated serine rich 2 like	0.73	0.07	9.81	2.33 × 10^−3^	3.70	1.30	2.81	3.75 × 10^−4^
XAF1	XIAP associated factor 1	121.6	37.89	3.21	1.62 × 10^−3^	79.70	17.80	4.47	3.75 × 10^−4^
HERC6	HECT and RLD domain containing E3 ubiquitin protein ligase family member 6	21.17	7.64	2.77	2.89 × 10^−3^	10.20	2.40	4.34	3.75 × 10^−4^
ASH2LP1	ASH2L pseudogene 1	14.35	5.48	2.62	1.02 × 10^−4^	2.00	0.50	4.25	7.00 × 10^−4^
ISG15	IFN-stimulated gene 15/ISG15 ubiquitin-like modifier	155.5	56.68	2.74	4.01 × 10^−3^	74.60	18.90	3.94	3.75 × 10^−4^
EPSTI1	Epithelial stromal interaction 1	52.02	17.97	2.89	2.89 × 10^−2^	70.20	22.80	3.08	3.75 × 10^−4^
PARP9	Poly (ADP-ribose) polymerase family member 9	39.69	14.84	2.67	9.63 × 10^−3^	38.60	9.30	4.14	3.75 × 10^−4^
EIF2AK2	Eukaryotic translation initiation factor 2 α kinase 2	22.54	9.65	2.34	1.51 × 10^−3^	31.70	14.10	2.24	3.75 × 10^−4^
PLSCR1	Phospholipid scramblase 1	32.86	13.63	2.41	4.71 × 10^−2^	39.80	11.60	3.44	3.75 × 10^−4^
GBP4	Guanylate binding protein 4	33.75	18.04	1.87	7.94 × 10^−2^	23.40	11.30	2.07	3.75 × 10^−4^
CD38	CD38 molecule	12.37	6.92	1.79	1.05 × 10^−3^	23.20	11.10	2.10	3.75 × 10^−4^
DTX3L	Deltex E3 ubiquitin ligase 3L	21.93	12.27	1.79	3.43 × 10^−2^	31.20	12.50	2.50	3.75 × 10^−4^
RNF213	Ring finger protein 213	9.49	5.82	1.63	1.28 × 10^−2^	92.10	53.50	1.72	3.75 × 10^−4^
IFITM1	Interferon Induced Transmembrane Protein 1	154.4	45.93	3.36	4.52 × 10^−3^	229.90	68.60	3.35	3.75 × 10^−4^
**Downregulated**									
PHYH	Phytanoyl-CoA 2-hydroxylase T cell differentiation and/or function of effector T cells.	3.100	4.83	0.64	9.00 × 10^−2^	3.20	4.90	0.65	3.96 × 10^−2^
IGHE	Immunoglobulin heavy constant epsilon	2.74	13.86	0.20	9.61 × 10^−2^	8.80	17.50	0.50	1.30 × 10^−3^
GALNTL6	Polypeptide N-acetylgalactosaminyl transferase like 6	0.03	0.69	0.05	9.61 × 10^−2^	0.20	1.90	0.10	3.75 × 10^−4^
PTPRG	Protein tyrosine phosphatase, receptor type G	0.01	0.13	0.09	4.10 × 10^−3^	0.50	2.00	0.23	3.75 × 10^−4^

FPKM, fragments per kilobase of transcript per million mapped reads. * Expression profiles based on Illumina RNA-seq. ** Benjamini–Hochberg-corrected *p*-value.

**Table 3:** Functional enrichment analysis results.

Ingenuity Canonical Pathways	−log (*p*-Value)	Molecules
Interferon Signaling	13.2	IFIT3, IFIT1, OAS1, MX1, STAT2, IFI35, IFITM1, ISG15
Role of Pattern Recognition Receptors in Recognition of Bacteria and Viruses	3.26	OAS1, OAS2, EIF2AK2, OAS3
Pyrimidine Deoxyribonucleotides De Novo Biosynthesis I	2.74	CMPK2, AK8
Salvage Pathways of Pyrimidine Ribonucleotides	2.62	CMPK2, AK8, EIF2AK2
Pyrimidine Ribonucleotides Interconversion	2.19	CMPK2, AK8
Pyrimidine Ribonucleotides De Novo Biosynthesis	2.15	CMPK2, AK8
Activation of IRF by Cytosolic Pattern Recognition Receptors	1.89	STAT2, ISG15

## Data Availability

All data relevant to the study are included in the article and are available on request from the corresponding author.

## Supplementary Materials

The following supporting information can be downloaded at https://www.mdpi.com/article/10.3390/genes15050628/s1: Table S1: differentially expressed genes; Table S2: disease and function annotation associated with dysregulated genes.
